# Implementing “Get Connected” Training in Long-term Care Settings during COVID

**DOI:** 10.1093/geroni/igaf122.4399

**Published:** 2025-12-31

**Authors:** Lené Levy-Storms, Eunice Kim, Susan Kohler

**Affiliations:** UCLA Luskin School of Public Affairs/Social Welfare, Los Angeles, California, United States; California State University, Los Angeles, California, United States; University of Southern California, Los Angeles, California, United States

## Abstract

A lack of emotionally attuned care occurs in long-term care (LTC) settings threatening residents’ quality of life, particularly those with cognitive deficits. This study offered “Get Connected (GC),” an evidence-based communication training intervention that teaches nine techniques for attaining emotional connections between direct care workers and residents using the EPIS framework (Exploration, Preparation, Implementation, Sustainment) with a train-the-trainer strategy. In the Exploration phase, this study recruited four of approximately 100 LTC facilities invited. In the Preparation phase, only 4 of these 5 sites ultimately participated. In the Implementation phase, two LTC facilities―each with one assisted living and one nursing home―offered GC with a total of 37 direct care workers over an eight-month period after respective accommodations. Entrenched organizational-level challenges, magnified by the lingering effects of the COVID-19 pandemic, posed sustained difficulties throughout implementation processes. Despite these issues, data analyses on post-training surveys with these 37 direct care workers indicated a high acceptability of GC strategies (95%). Additional results from 6 separate focus groups with three stakeholder types (total N = 34: 11 administrators, 11 staff, 12 residents) reaffirmed GC training as a valuable refresher and emphasized the importance of reinforcement. Additional results from both survey and focus group respondents supporting wider uptake in LTC settings (99%) suggest promise for the Sustainment phase. Implications will require concerted efforts among LTC stakeholders at multiple levels, including policy makers, administration, and the care workforce, to transform LTC training infrastructure to prioritize humanity during care.

